# Supplementary material for: Translation initiation by the Kozak mRNA sequence is based on a conformational readout on the ribosome
Source: Nat Commun. 2026 Jul 8;17:8467. doi: 10.1038/s41467-026-73969-2 (PMC13478227; doi:10.1038/s41467-026-73969-2)
Supplement: Supplementary file 2 [file 41467_2026_73969_MOESM2_ESM.pdf]

nature portfolio

Corresponding author(s): Bruno Klaholz

Last updated by author(s): Apr 30, 2026

# Reporting Summary

Nature Portfolio wishes to improve the reproducibility of the work that we publish. This form provides structure for consistency and transparency in reporting. For further information on Nature Portfolio policies, see our Editorial Policies and the Editorial Policy Checklist.

## Statistics

For all statistical analyses, confirm that the following items are present in the figure legend, table legend, main text, or Methods section.

| n/a | Confirmed | |
|---|---|---|
| ☐ | ☒ | The exact sample size (*n*) for each experimental group/condition, given as a discrete number and unit of measurement |
| ☐ | ☒ | A statement on whether measurements were taken from distinct samples or whether the same sample was measured repeatedly |
| ☐ | ☒ | The statistical test(s) used AND whether they are one- or two-sided<br>*Only common tests should be described solely by name; describe more complex techniques in the Methods section.* |
| ☒ | ☐ | A description of all covariates tested |
| ☒ | ☐ | A description of any assumptions or corrections, such as tests of normality and adjustment for multiple comparisons |
| ☐ | ☒ | A full description of the statistical parameters including central tendency (e.g. means) or other basic estimates (e.g. regression coefficient) AND variation (e.g. standard deviation) or associated estimates of uncertainty (e.g. confidence intervals) |
| ☐ | ☒ | For null hypothesis testing, the test statistic (e.g. *F*, *t*, *r*) with confidence intervals, effect sizes, degrees of freedom and *P* value noted<br>*Give P values as exact values whenever suitable.* |
| ☒ | ☐ | For Bayesian analysis, information on the choice of priors and Markov chain Monte Carlo settings |
| ☒ | ☐ | For hierarchical and complex designs, identification of the appropriate level for tests and full reporting of outcomes |
| ☒ | ☐ | Estimates of effect sizes (e.g. Cohen's *d*, Pearson's *r*), indicating how they were calculated |

*Our web collection on statistics for biologists contains articles on many of the points above.*

## Software and code

Policy information about availability of computer code

| | |
|---|---|
| Data collection | EPU, SerialEM |
| Data analysis | Cryo-EM image processing software: RELION v. 3.0 and v.4.0.0 Phenix 1.21, Coot 0.9.8.93, cryoSPARC v. 4.1.1, Gautomatch , MotionCor2 v1.6.3, VUE |

For manuscripts utilizing custom algorithms or software that are central to the research but not yet described in published literature, software must be made available to editors and reviewers. We strongly encourage code deposition in a community repository (e.g. GitHub). See the Nature Portfolio guidelines for submitting code & software for further information.

## Data

Policy information about availability of data

All manuscripts must include a data availability statement. This statement should provide the following information, where applicable:

- Accession codes, unique identifiers, or web links for publicly available datasets
- A description of any restrictions on data availability
- For clinical datasets or third party data, please ensure that the statement adheres to our policy

Atomic coordinates and cryo-EM maps have been deposited in the Protein Data Bank (PDB) and Electron Microscopy Data Bank (EMDB) under accession codes pdb_000028ZX [https://dx.doi.org/10.2210/pdb28zx/pdb] and EMD-57009 [https://www.ebi.ac.uk/emdb/EMD-57009] (PDB ID and composite map for the Kozak_1 48S PIC), pdb_000028ZY [https://dx.doi.org/10.2210/pdb28zy/pdb] and EMD-57010 [https://www.ebi.ac.uk/emdb/EMD-57010]  (PDB ID and composite map for

the Kozak_2 48S PIC), pdb_000028ZV [https://dx.doi.org/10.2210/pdb28zv/pdb] and EMD-57005 [https://www.ebi.ac.uk/emdb/EMD-57005] (PDB ID and composite map for the non-Kozak 48S PIC) and pdb_000028ZU [https://dx.doi.org/10.2210/pdb28zu/pdb] and EMD-57004 [https://www.ebi.ac.uk/emdb/EMD-57004] (PDB ID and composite map for the Kozak 48S PIC containing the eIF1A W70A mutant). Additionally focused refined maps were deposited for the 40S ribosomal subunit body with mRNA and eIF1A (EMD-57017 [https://www.ebi.ac.uk/emdb/EMD-57017]), the 40S ribosomal subunit head region with TC (EMD-57018 [https://www.ebi.ac.uk/emdb/EMD-57018]) and eIF3 (EMD-57019 [https://www.ebi.ac.uk/emdb/EMD-57019]), respectively for the Kozak_1 48S PIC; for the 40S ribosomal subunit body with mRNA and eIF1A (EMD-57015 [https://www.ebi.ac.uk/emdb/EMD-57015]), and 40S ribosomal subunit head region with TC (EMD-57014 [https://www.ebi.ac.uk/emdb/EMD-57014]) and eIF3 (EMD-57013 [https://www.ebi.ac.uk/emdb/EMD-57013]), respectively for the Kozak_2 48S PIC; for the 40S ribosomal subunit body with mRNA and eIF1A (EMD-57011 [https://www.ebi.ac.uk/emdb/EMD-57011]), and 40S ribosomal subunit head region with TC (EMD-56980 [https://www.ebi.ac.uk/emdb/EMD-56980]), respectively for the non-Kozak 48S PIC; and for the 40S ribosomal subunit body with mRNA and eIF1A (EMD-56982 [https://www.ebi.ac.uk/emdb/EMD-56982]), and 40S ribosomal subunit head region with TC (EMD-56983 [https://www.ebi.ac.uk/emdb/EMD-56983]), respectively for the Kozak 48S PIC with the eIF1A W70A mutant. The maps from the last global refinement of the 48S PICs before the focused refinements were deposited under accession codes EMD-57020 [https://www.ebi.ac.uk/emdb/EMD-57020] (Kozak_1 48S PIC), EMD-57016 [https://www.ebi.ac.uk/emdb/EMD-57016] (Kozak_2 48S PIC), EMD-57012 [https://www.ebi.ac.uk/emdb/EMD-57012] (non-Kozak 48S PIC) and EMD-56981 [https://www.ebi.ac.uk/emdb/EMD-56981] (Kozak 48S PIC with the eIF1A W70A mutant). The data generated for Figs. 1a, 1b, 3d and Supplementary Fig. 11b&c are available as source data along with this manuscript.

## Research involving human participants, their data, or biological material

Policy information about studies with human participants or human data. See also policy information about sex, gender (identity/presentation), and sexual orientation and race, ethnicity and racism.

| | |
|---|---|
| Reporting on sex and gender | N/A |
| Reporting on race, ethnicity, or other socially relevant groupings | N/A |
| Population characteristics | N/A |
| Recruitment | N/A |
| Ethics oversight | N/A |

Note that full information on the approval of the study protocol must also be provided in the manuscript.

# Field-specific reporting

Please select the one below that is the best fit for your research. If you are not sure, read the appropriate sections before making your selection.

☒ Life sciences ☐ Behavioural & social sciences ☐ Ecological, evolutionary & environmental sciences

For a reference copy of the document with all sections, see nature.com/documents/nr-reporting-summary-flat.pdf

# Life sciences study design

All studies must disclose on these points even when the disclosure is negative.

| | |
|---|---|
| Sample size | N/A |
| Data exclusions | N/A |
| Replication | N/A |
| Randomization | N/A |
| Blinding | N/A |

# Reporting for specific materials, systems and methods

We require information from authors about some types of materials, experimental systems and methods used in many studies. Here, indicate whether each material, system or method listed is relevant to your study. If you are not sure if a list item applies to your research, read the appropriate section before selecting a response.

## Materials & experimental systems

| n/a | Involved in the study |
|---|---|
| ☒ | ☐ Antibodies |
| ☐ | ☒ Eukaryotic cell lines |
| ☒ | ☐ Palaeontology and archaeology |
| ☒ | ☐ Animals and other organisms |
| ☒ | ☐ Clinical data |
| ☒ | ☐ Dual use research of concern |
| ☒ | ☐ Plants |

## Methods

| n/a | Involved in the study |
|---|---|
| ☒ | ☐ ChIP-seq |
| ☒ | ☐ Flow cytometry |
| ☒ | ☐ MRI-based neuroimaging |

# Eukaryotic cell lines

Policy information about cell lines and Sex and Gender in Research

| | |
|---|---|
| Cell line source(s) | HeLa, HEK 293T and hTERT RPE1 cell lines at IGBMC PluriCell East platform |
| Authentication | Cells were tested by polymerase chain reaction for Mycoplasma and certified as free of Mycoplasma. |
| Mycoplasma contamination | Cells were tested by polymerase chain reaction for Mycoplasma and certified as free of Mycoplasma. |
| Commonly misidentified lines (See ICLAC register) | *Name any commonly misidentified cell lines used in the study and provide a rationale for their use.* |

# Plants

| | |
|---|---|
| Seed stocks | N/A |
| Novel plant genotypes | N/A |
| Authentication | N/A |
