# Supplementary material for: Translation initiation by the Kozak mRNA sequence is based on a conformational readout on the ribosome
Source: Nat Commun. 2026 Jul 8;17:8467. doi: 10.1038/s41467-026-73969-2 (PMC13478227; doi:10.1038/s41467-026-73969-2)
Supplement: Supplementary file 3 [file 41467_2026_73969_MOESM3_ESM.pdf]

nature portfolio

Peer Review File

Translation initiation by the Kozak mRNA sequence is based on a conformational readout on the ribosome

Corresponding Author: Dr Bruno Klaholz

This file contains all reviewer reports in order by version, followed by all author rebuttals in order by version.

Version 0:

Reviewer comments:

Reviewer #1

(Remarks to the Author)
This manuscript describes the structures of the eucaryotic initiation complex on the 40S ribosomal subunit upon binding the initiator AUG sequence (and its surrounding bases) with proper and improper Kozak sequences, and with a cofactor mutant structure. The structures are well-determined to 2.8-3Å resolution using 30-40000 particle images for each of the three structures, which is typical for ribosome structures. The modeling was done carefully and was mostly easily validated (but see below). The manuscript is well-written and the structural features of each of the sites is described in great detail. I especially appreciated that the rearrangements relative to the proper Kozak structure were able to be described with an alternate structure, rather than simply disappearing.
I have a few comments.
1. The density maps, along with the models, are provided for some regions of the structures, and appeared to have the appropriate morphology for the stated resolution. More examples would have been appreciated.
2. Alternatively, or even better, would be to quote the Q-score for each of the residues/bases, or groups of bases (eg, the region from -3 to +4-7 of the start codon) for the different Kozak/non-Kozak structures.
3. Q-scores, found in the validation reports*, should be reported in the body of the manuscript. For the three structures, the Q-scores appear to be almost exactly as expected for structures at 2.8-3Å resolution (around 0.55), so these are solidly-modeled structures overall. This makes the local Q-score around the residues/bases discussed even more important for validating their interpretations and their confidence in them. Per-atom, per-residue and per-sequence Q-scores are easily calculated in Chimera.
4. *Regarding the Validation Reports, the ones provided to this reviewer are preliminary and explicitly state they are not to be used for manuscript review. Please deposit the maps and models so there is a PDB and EMD number assigned to each and a corresponding validation report for each. Submitting the final reports will make it possible to determine which validation report goes with which PDB/EMDB deposition. I find the Validation Reports to be critical for evaluating structures.

Reviewer #2

(Remarks to the Author)
In this work, Loeffelholz et al. report multiple cryo-EM structures of human 48S preinitiation complexes (PICs) captured on mRNAs with start codon AUG in Kozak and non-Kozak contexts. The comparison of the complexes captured in the two contexts bring out the mode of recognition of the Kozak sequences in eukaryotic 48S PICs. This study describes the Kozak context and its recognition in details. A thorough structural analysis of previously reported structures and the PICs reported in this work is presented.
This work brings out the role of 'rRNA-based environment' for the interaction of Kozak sequences of mRNA with the PICs and the eIFs, eIF2α and eIF1A, provide additional selectivity for this recognition. Furthermore, Kozak-rRNA interaction relies on base stacking instead of the complementary base pairing observed in the case Shine Dalgarno (SD) / anti-SD in bacteria. Moreover, the authors suggest that in both -3 and +4 positions of Kozak sequence the nucleotides are stabilized as entire base triplets. Although it is the first nucleotide of each triplet (-3 & +4) that provides specificity, entire triplet codons are stabilized. The study also brings out the role of conserved Arg/Lys residues of eIF2α & Trp70 of eIF1A.
However, I have few comments /queries that I would like to the authors to address before publication of this work.

1. The validation reports submitted are 'NOT for manuscript review'. Hence proper validation reports need to be submitted.

Also, the maps & atomic coordinates are not available for review. Without these, it is difficult to comment on the details reported in this manuscript.
2. Why authors added/supplemented eIF1A to 48S PIC before grid freezing?
3. How was the sequence of mRNAs in 48S PIC confirmed especially for non-Kozak & W70A PICs confirmed since the local resolution of mRNAs is low?
4. Was the synthetic mRNA used for 48S PIC capped or uncapped? What is known about efficiency of uncapped mRNA outcompeting cellular capped mRNAs?
5. Page 3 line 47-48: “preferably a G at the +4 position with the exception of fungi that have a bias for U”. The authors have referred to an earlier study. However, a recent study (Hernandez et al 2024; reference 45) shows that an A at position +4 is preferred in Fungi.
6. Base stacking in -3 to -1 region of Kozak was observed and reported in the 2018 study of yeast 48S PIC (Figure 6C of Llacer et al 2018; Reference 39). The authors should add this yeast 48S PIC in the structural analysis presented here.

Reviewer #3

(Remarks to the Author)
In their article, von Loeffelholz and co-authors studied Kozak sequence recognition in the context of the 48S PIC from human. The authors first used in vitro and in vivo translation assays with mRNAs having various Kozak sequences from the perfect Kozak sequence to sequences containing pyrimidines in -3 and +4. The results led the authors to select two mRNA sequences either containing a perfect Kozak context “ACCAUGGAA” or a “non-Kozak” “UUUAUGUUA” one to reconstitute 48S PICs. To this aim, the authors implemented a complex isolation procedure using HeLa cell extracts blocked with GMPPNP mixed with large amounts of the mRNAs under study. After a sucrose gradient fractionation step, the 48S PIC fraction was supplemented with eIF1A in excess to circumvent initiation factor dissociation during preparation. The 48S PICs were then used for cryo-EM analysis. 3D classification and sorting techniques enabled them to address conformational variability. Two conformations were obtained with the perfect Kozak mRNA and one with the non-Kozak mRNA.
The main results show that adenosine -3 is stabilized by a pi-stacking interaction with R55 of the D1 loop of eIF2α. This interaction favors the interaction of the pre-codon with 2 conserved guanines of the rRNA forming a G-clamp structure. However, when a pyrimidine is present at -3, R55 does not interact with it and the positioning of the pre-codon appears to be more dynamic. Furthermore, a +4 purine favors a very nice network of pi-stacking interactions from +4 to +8 bases. This network of interaction involves W70 of eIF1A and the decoding base A1825. K67 of eIF1A participates in the mechanism through an interaction with +4 of the mRNA and G626 of the rRNA. Comparison of perfect Kozak and non-Kozak 48S PIC structures suggests an induced fit mechanism of rRNA and eIF1A dependent on the +4 purine of the mRNA. This induced-fit mechanism is also supported by the analysis of the second conformation of the 48S PIC observed in the presence of perfect Kozak mRNA.
Finally, the authors also studied the impact of two eIF1A mutations, W70A and R66A/K68A. In vitro translation assays and the cryo-EM structure of a 48S PIC obtained in the presence of eIF1A W70A showed that the 66-68 region of eIF1A is important for the induced fit mechanism while W70 is not essential. The authors also carefully compared their results with previously determined structures of Mammalian 48S PICs (Extended Data 7 and 8).
To conclude, the present work shows how -3 and +4 purines contribute to mRNA stabilization at the P site through an induced fit mechanism, thereby answering a long-standing question about the role of purines -3 and +4 in the Kozak sequence. The experiments are robust. The article is well written and provides new conceptual information about Kozak recognition. I therefore strongly recommend its publication in Nature communications.

I have a few minor comments and remarks to make:
Comments :
-In the last sentence of the Abstract, I would remove « instead of relying on base pairing as in bacteria » that is not clear enough at this stage. The comparison between the interaction of the Kozak sequence with rRNA and the SD :antiSD interaction is better explained in lines 364-365.

-Kozak_1 and Kozak_2 are equally represented in terms of particle count. Why is Kozak_1 not more represented when it is supposed to be more stable (see line 458)? One possibility is that the two conformations are equally stable but that the Kozak_1 conformation is more favorable for the eIF5B step. Here, since PIC 48S is blocked by GMPPNP, the subsequent eIF5B step cannot occur, leading to an equilibrium between the two Kozak conformations. In standard translation initiation conditions, eIF5B binding would occur more easily with Kozak_1 which will shift the equilibrium in favor of Kozak_1.
In this view, authors may comment on the position of eIF1A. Is the position of eIF1A in Kozak_1 closer to that observed in a 40S:mRNA:tRNA:eIF1A:eIF5B complex, which could suggest that Kozak_1 is might be more favorable to the eIF5B step? In addition, the authors did not comment on the position and interactions of the N-terminal tail of eIF1A in the P site for Kozak_1 and Kozak_2. Is there any difference between the two conformations ?

Minor points :
-line 40 add « approximately » before « 8 nucleotides ».
-line 48 : fungi have a bias for U at +4 position of the Kozak. Does this study provide any evidence to explain this preference that could be discussed ?
-lines 114 to 119 : description of the head conformations of the Kozak complex should be clarified perhaps by first mentioning that closed head conformations were selected and that one of the two is slightly more open than the other. The comparison with the open state may be misleading. Concerning this point, are there any clues to explain why the Kozak_1 conformation is slightly more open than the Kozak_2 conformation?
-line 139 : add initiation to « translational state ».

-line 236 ; replace 48S PIC by mRNA.
-lines 298-299 ; the sentence in parentheses is unclear.
-line 452 replace unproductive by less productive.
-line 483 please change conformal by conformational.
-add Panel labels in the legend of Fig.4.
-Accession numbers of the PDB files and Cryo-EM maps have to be indicated in the manuscript.
-Change Extended Figures into Supplementary Figures and correct the text accordingly.

Emmanuelle Schmitt

Version 1:

Reviewer comments:

Reviewer #1

(Remarks to the Author)
The authors have addressed all my concerns. I appreciate their attention to the PDB Validation Reports.

Reviewer #2

(Remarks to the Author)
The authors have addressed all the comments and I am satisfied with the answers provided.

Reviewer #3

(Remarks to the Author)
The authors have provided satisfactory answers to all reviewers' questions. I therefore recommend the publication of the article in Nature Communications.

Open Access This Peer Review File is licensed under a Creative Commons Attribution 4.0 International License, which permits use, sharing, adaptation, distribution and reproduction in any medium or format, as long as you give appropriate credit to the original author(s) and the source, provide a link to the Creative Commons license, and indicate if changes were made.
In cases where reviewers are anonymous, credit should be given to 'Anonymous Referee' and the source.
The images or other third party material in this Peer Review File are included in the article’s Creative Commons license, unless indicated otherwise in a credit line to the material. If material is not included in the article’s Creative Commons license and your intended use is not permitted by statutory regulation or exceeds the permitted use, you will need to obtain permission directly from the copyright holder.
To view a copy of this license, visit https://creativecommons.org/licenses/by/4.0/

REVIEWER COMMENTS

Reviewer #1 (Remarks to the Author):

This manuscript describes the structures of the eucaryotic initiation complex on the 40S ribosomal subunit upon binding the initiator AUG sequence (and its surrounding bases) with proper and improper Kozak sequences, and with a cofactor mutant structure. The structures are well-determined to 2.8-3Å resolution using 30-40000 particle images for each of the three structures, which is typical for ribosome structures. The modeling was done carefully and was mostly easily validated (but see below). The manuscript is well-written and the structural features of each of the sites is described in great detail. I especially appreciated that the rearrangements relative to the proper Kozak structure were able to be described with an alternate structure, rather than simply disappearing.

We would like to thank the reviewer for this very positive and fair feedback. We have addressed all remaining concerns in the revised manuscript as detailed below.

I have a few comments.
1. The density maps, along with the models, are provided for some regions of the structures, and appeared to have the appropriate morphology for the stated resolution. More examples would have been appreciated.

We have added more examples along with corresponding Q-scores in the newly created Supplementary Fig. 5.

2. Alternatively, or even better, would be to quote the Q-score for each of the residues/bases, or groups of bases (eg, the region from -3 to +4-7 of the start codon) for the different Kozak/non-Kozak structures.

As suggested, we have added the per chain and per residue Q-scores for important nucleotides/residues (i.e. 18S rRNA, eIF2α, eIF1A and mRNA) in the newly created Supplementary Fig. 5.

3. Q-scores, found in the validation reports, should be reported in the body of the manuscript. For the three structures, the Q-scores appear to be almost exactly as expected for structures at 2.8-3Å resolution (around 0.55), so these are solidly-modeled structures overall. This makes the local Q-score around the residues/bases discussed even more important for validating their interpretations and their confidence in them. Per-atom, per-residue and per-sequence Q-scores are easily calculated in Chimera.*

As suggested, we have now added the overall Q scores provided by the PDB validation reports in Table 2 and the per residue and per chain Q-scores (dependent on the estimated resolution at sigma 0.4) of areas of interest calculated per chain Q-scores from the validation reports and per residue Q-scores calculated with MapQ v.2.9.7) in the new Supplementary Fig. 5.

*4. *Regarding the Validation Reports, the ones provided to this reviewer are preliminary and explicitly state they are not to be used for manuscript review. Please deposit the maps and models so there is a PDB and EMD number assigned to each and a corresponding validation report for each. Submitting the final reports will make it possible to determine which validation report goes with which PDB/EMDB deposition. I find the Validation Reports to be critical for evaluating structures.*

We have now deposited the refined map and models to the EMDB / PDB, included the PDB IDs and

EMDB codes in Table 1 and 2 and in the Data availability section of the manuscript and uploaded all final validation reports to the revised manuscript.

Reviewer #2 (Remarks to the Author):

In this work, Loeffelholz et al. report multiple cryo-EM structures of human 48S preinitiation complexes (PICs) captured on mRNAs with start codon AUG in Kozak and non-Kozak contexts. The comparison of the complexes captured in the two contexts bring out the mode of recognition of the Kozak sequences in eukaryotic 48S PICs. This study describes the Kozak context and its recognition in details. A thorough structural analysis of previously reported structures and the PICs reported in this work is presented.
This work brings out the role of 'rRNA-based environment' for the interaction of Kozak sequences of mRNA with the PICs and the eIFs, eIF2α and eIF1A, provide additional selectivity for this recognition. Furthermore, Kozak-rRNA interaction relies on base stacking instead of the complementary base pairing observed in the case Shine Dalgarno (SD) / anti-SD in bacteria. Moreover, the authors suggest that in both -3 and +4 positions of Kozak sequence the nucleotides are stabilized as entire base triplets. Although it is the first nucleotide of each triplet (-3 & +4) that provides specificity, entire triplet codons are stabilized. The study also brings out the role of conserved Arg/Lys residues of eIF2α & Trp70 of eIF1A.

However, I have few comments /queries that I would like to the authors to address before publication of this work.

We would like to thank the reviewer for this very positive and fair feedback. We have addressed all remaining points in the revised manuscript as detailed below.

1. The validation reports submitted are 'NOT for manuscript review'. Hence proper validation reports need to be submitted. Also, the maps & atomic coordinates are not available for review. Without these, it is difficult to comment on the details reported in this manuscript.

We had provided maps and atomic coordinates for initial review but there was a problem with the journal's website apparently. In any case, we submitted the refined map and models to the PDB and uploaded final validation reports as part of the revised manuscript. EMDB and PDB IDs are now also included in Tables 1 and 2 and in the Data availability section of the manuscript.

2. Why authors added/supplemented eIF1A to 48S PIC before grid freezing?

The rational for our approach is that eIF1A tends to dissociate from the complex during sucrose gradient centrifugation if not cross-linked (which we did not do); we specified this now more clearly in the methods section.

3. How was the sequence of mRNAs in 48S PIC confirmed especially for non-Kozak & W70A PICs confirmed since the local resolution of mRNAs is low?

At the given resolution, the presence of purine or pyrimidine in the sequence from -3 to +3 can be confidently confirmed in all cases.
For example, the density for U-3 in the non-Kozak PIC is clearly smaller than that of the A-3 in the Kozak PIC. The presence of an GAA codon in the A-site of the W70A PIC can also be clearly assigned.

As for the other complexes, using an excess of mRNA in the cell extracts ensured that endogenous mRNA sequences became a minor species (as described in the methods section).

4. Was the synthetic mRNA used for 48S PIC capped or uncapped? What is known about efficiency of uncapped mRNA outcompeting cellular capped mRNAs?

In wheat germ extract the efficiency of using uncapped mRNA is about 10-50% in comparison to capped mRNA (PMID:2246243 Fletcher et al. JBC, 1990). We used synthetic uncapped mRNA in HeLa cell extracts. Our structures show clearly (clear difference in nucleotide density of U-3 vs. A-3) that the synthetic uncapped mRNA binds to the 43S PIC in the cell extract efficiently at the high excess we used. In our opinion, to evaluate and compare the role of individual Kozak residues it is easier to use non-capped mRNA.

5. Page 3 line 47-48: "preferably a G at the +4 position with the exception of fungi that have a bias for U". The authors have referred to an earlier study. However, a recent study (Hernandez et al 2024; reference 45) shows that an A at position +4 is preferred in Fungi.

Indeed, we have corrected that in the text now to cite both: " …(preferably a G at the +4 position with the exception of fungi that have a bias for A[10] and in some cases U[11])…".

6. Base stacking in -3 to -1 region of Kozak was observed and reported in the 2018 study of yeast 48S PIC (Figure 6C of Llacer et al 2018; Reference 39). The authors should add this yeast 48S PIC in the structural analysis presented here.

We have added new panels for the PDB ID 6fyy (Llacer *et al.*, Elife 2018) in Supplementary figures 8 and 9 (previously 7 and 8, renumbered due to the newly created Supplementary Fig. 5).

Reviewer #3 (Remarks to the Author):

In their article, von Loeffelholz and co-authors studied Kozak sequence recognition in the context of the 48S PIC from human. The authors first used in vitro and in vivo translation assays with mRNAs having various Kozak sequences from the perfect Kozak sequence to sequences containing pyrimidines in -3 and +4. The results led the authors to select two mRNA sequences either containing a perfect Kozak context "ACCAUGGAA" or a "non-Kozak" "UUUAUGUUA" one to reconstitute 48S PICs. To this aim, the authors implemented a complex isolation procedure using HeLa cell extracts blocked with GMPPNP mixed with large amounts of the mRNAs under study. After a sucrose gradient fractionation step, the 48S PIC fraction was supplemented with eIF1A in excess to circumvent initiation factor dissociation during preparation. The 48S PICs were then used for cryo-EM analysis. 3D classification and sorting techniques enabled them to address conformational variability. Two conformations were obtained with the perfect Kozak mRNA and one with the non-Kozak mRNA.
The main results show that adenosine -3 is stabilized by a pi-stacking interaction with R55 of the D1 loop of eIF2α. This interaction favors the interaction of the pre-codon with 2 conserved guanines of the rRNA forming a G-clamp structure. However, when a pyrimidine is present at -3, R55 does not interact with it and the positioning of the pre-codon appears to be more dynamic. Furthermore, a +4 purine favors a very nice network of pi-stacking interactions from +4 to +8 bases. This network of interaction involves W70 of eIF1A and the decoding base A1825. K67 of eIF1A participates in the mechanism through an interaction with +4 of the mRNA and G626 of the rRNA. Comparison of perfect Kozak and non-Kozak 48S PIC structures suggests an induced fit mechanism of rRNA and eIF1A dependent on the +4 purine of the mRNA. This induced-fit mechanism is also supported by the

analysis of the second conformation of the 48S PIC observed in the presence of perfect Kozak mRNA. Finally, the authors also studied the impact of two eIF1A mutations, W70A and R66A/K68A. In vitro translation assays and the cryo-EM structure of a 48S PIC obtained in the presence of eIF1A W70A showed that the 66-68 region of eIF1A is important for the induced fit mechanism while W70 is not essential. The authors also carefully compared their results with previously determined structures of Mammalian 48S PICs (Extended Data 7 and 8).
To conclude, the present work shows how -3 and +4 purines contribute to mRNA stabilization at the P site through an induced fit mechanism, thereby answering a long-standing question about the role of purines -3 and +4 in the Kozak sequence. The experiments are robust. The article is well written and provides new conceptual information about Kozak recognition. I therefore strongly recommend its publication in Nature communications.

We would like to thank the reviewer for this very positive and fair feedback. We have addressed all remaining points in the revised manuscript as detailed below.

I have a few minor comments and remarks to make:
Comments :
-In the last sentence of the Abstract, I would remove « instead of relying on base pairing as in bacteria » that is not clear enough at this stage. The comparison between the interaction of the Kozak sequence with rRNA and the SD :antiSD interaction is better explained in lines 364-365.

At the level of the abstract, this is meant as a summary of the recognition mechanism / concept (which is different between species), while details are indeed explained later. We prefer to keep it so that people immediately see that the molecular mechanism discussed here is fundamentally different from the well-known SD-dependent translation initiation mechanism.

-Kozak_1 and Kozak_2 are equally represented in terms of particle count. Why is Kozak_1 not more represented when it is supposed to be more stable (see line 458)? One possibility is that the two conformations are equally stable but that the Kozak_1 conformation is more favorable for the eIF5B step. Here, since PIC 48S is blocked by GMPPNP, the subsequent eIF5B step cannot occur, leading to an equilibrium between the two Kozak conformations. In standard translation initiation conditions, eIF5B binding would occur more easily with Kozak_1 which will shift the equilibrium in favor of Kozak_1.
In this view, authors may comment on the position of eIF1A. Is the position of eIF1A in Kozak_1 closer to that observed in a 40S:mRNA:tRNA:eIF1A:eIF5B complex, which could suggest that Kozak_1 is might be more favorable to the eIF5B step? In addition, the authors did not comment on the position and interactions of the N-terminal tail of eIF1A in the P site for Kozak_1 and Kozak_2. Is there any difference between the two conformations ?

This is what we first thought too, but the eIF5B conformation seen in previous reports would clash with the eIF1A OB domain in the Kozak_1 conformation (this may also depend on the eIF5B conformation and eIF5 presence, there are yet several steps to be understood before eIF5B binds). It is possible that the Kozak_1 conformation first stabilizes start codon recognition and subsequently changes conformation allowing factors to bind. More structures and future research are necessary. However, our data show that eIF1A does adopt different conformations, which has an effect on the mRNA structure and very likely on the stability of the 48S PIC, as suggested by the referee.
The N-terminus of eIF1A can only be confidently fit from Gly6 onwards. Residues 1-5 are not very stable in all structures. For this reason, we show the N-terminus (residues 1-5) in transparency in Figures 3 and 4. Residues 1-5 of the Kozak_2 complex seems slightly more stable, but any conclusions based on the N-terminus of eIF1A would be too imprecise at this point. Stabilization of the tRNA-mRNA mini-helix by the eIF1A N-terminus is now mentioned in the manuscript: "…rRNA

($m^1acp^3\Psi 1248$) around the codon-anticodon mini-helix (**Fig. 1D**; see methods) that is further stabilized by the N-terminal tail of eIF1A (residues 7-9) similarly as observed before[14,15,22,26,39,40].".

Minor points:
-line 40 add « approximately » before « 8 nucleotides ».

We have modified the text accordingly.

-line 48 : fungi have a bias for U at +4 position of the Kozak. Does this study provide any evidence to explain this preference that could be discussed ?

This is difficult to say as the overall sequence context can be quite variable in yeast. Clearly, a U in the +4 position is not recognized by neither A1825 nor Trp70 of eIF1A as our structures and Llacer *et al.* (2018) show; hence, how a U+4 in yeast would enhance translation is difficult to tell, also because the -3 region is probably important there also (it has an AAA). An A+4 is actually more common, as we have now added in the introduction.

-lines 114 to 119 : description of the head conformations of the Kozak complex should be clarified perhaps by first mentioning that closed head conformations were selected and that one of the two is slightly more open than the other. The comparison with the open state may be misleading. Concerning this point, are there any clues to explain why the Kozak_1 conformation is slightly more open than the Kozak_2 conformation?

We have clarified the text as follows:

"These complexes were analysed by high-resolution cryo-EM and advanced structure sorting techniques to firstly select all 48S PICs with a "closed" head conformation present in the sample, followed by sub-classification of fine differences in 40S head conformations to address conformational variability[27–35], revealing two different "closed" head conformations for all data sets (**Supplementary Fig. 1**).

Closer analysis of the Kozak complex revealed two "closed" conformations of the 48S PIC differing in the degree of head closure,…"

To our understanding the Kozak sequence can be better recognized before the head is completely closed. Complete closure of the head is likely occurring after Kozak sequence recognition.

-line 139 : add initiation to « translational state ».
Done.

-line 236 ; replace 48S PIC by mRNA.
Done.

-lines 298-299 ; the sentence in parentheses is unclear.
We have clarified the text as follows: "(specifically for Kozak_1 versus Kozak_2, not seen for non-Kozak)".

-line 452 replace unproductive by less productive.
Done.

-line 483 please change conformal by conformational.
Corrected.

-add Panel labels in the legend of Fig.4.
Done.

-Accession numbers of the PDB files and Cryo-EM maps have to be indicated in the manuscript.
We submitted the refined cryo-EM maps and models to the PDB and added the PDB and EMDB codes in Table 1 and 2 as well as in the section Data availability of the manuscript. The EMDB codes are now also stated in Supplementary Fig.1.

-Change Extended Figures into Supplementary Figures and correct the text accordingly.
Done.

Emmanuelle Schmitt
